# Mesenchymal Stromal Cell on Liver Decellularised Extracellular Matrix for Tissue Engineering

**DOI:** 10.3390/biomedicines10112817

**Published:** 2022-11-04

**Authors:** Stefania Croce, Lorenzo Cobianchi, Tamara Zoro, Francesca Dal Mas, Antonia Icaro Cornaglia, Elisa Lenta, Gloria Acquafredda, Annalisa De Silvestri, Maria Antonietta Avanzini, Livia Visai, Szandra Brambilla, Giovanna Bruni, Giulia Di Gravina, Andrea Pietrabissa, Luca Ansaloni, Andrea Peloso

**Affiliations:** 1 Department of Clinical, Surgical, Diagnostic & Pediatric Sciences, University of Pavia, 27100 Pavia, Italy; 2 Department of General Surgery, Fondazione IRCCS Policlinico San Matteo, 27100 Pavia, Italy; 3 Department of Management, Ca’ Foscari University of Venice, 30100 Venice, Italy; 4 Histology & Embryology Unit, Department of Public Health, Experimental Medicine & Forensic, University of Pavia, 27100 Pavia, Italy; 5 Immunology and Transplantation Laboratory, Cell Factory, Pediatric Hematology Oncology, Fondazione IRCCS Policlinico San Matteo, 27100 Pavia, Italy; 6 Biometry & Clinical Epidemiology, Scientific Direction, Fondazione IRCCS Policlinico San Matteo, 27100 Pavia, Italy; 7 Center for Health Technologies (CHT), Department of Molecular Medicine, INSTM UdR of Pavia, University of Pavia, Viale Taramelli 3/b, 27100 Pavia, Italy; 8 Medicina Clinica-Specialistica, UOR5 Laboratorio di Nanotecnologie, ICS Maugeri, IRCCS, Via S. Boezio 28, 27100 Pavia, Italy; 9 CSGI Department of Physical Chemistry M Rolla, 27100 Pavia, Italy; 10 Department of Industrial and Information Engineering, University of Pavia, 27100 Pavia, Italy; 11 Hepatology and Transplantation Laboratory, Department of Surgery, Faculty of Medicine, University of Geneva, 1205 Geneva, Switzerland; 12 Divisions of Abdominal and Transplantation Surgery, Department of Surgery, Geneva University Hospitals, 1205 Geneva, Switzerland

**Keywords:** mesenchymal stromal cells, bioscaffold, decellularisation, extracellular matrix, recellularisation, tissue engineering

## Abstract

Background: In end-stage chronic liver disease, transplantation represents the only curative option. However, the shortage of donors results in the death of many patients. To overcome this gap, it is mandatory to develop new therapeutic options. In the present study, we decellularised pig livers and reseeded them with allogeneic porcine mesenchymal stromal cells (pMSCs) to understand whether extracellular matrix (ECM) can influence and/or promote differentiation into hepatocyte-like cells (HLCs). Methods: After decellularisation with SDS, the integrity of ECM-scaffolds was examined by histological staining, immunofluorescence and scanning electron microscope. DNA quantification was used to assess decellularisation. pMSCs were plated on scaffolds by static seeding and maintained in in vitro culture for 21 days. At 3, 7, 14 and 21 days, seeded ECM scaffolds were evaluated for cellular adhesion and growth. Moreover, the expression of specific hepatic genes was performed by RT-PCR. Results: The applied decellularisation/recellularisation protocol was effective. The number of seeded pMSCs increased over the culture time points. Gene expression analysis of seeded pMSCs displayed a weak induction due to ECM towards HLCs. Conclusions: These results suggest that ECM may address pMSCs to differentiate in hepatocyte-like cells. However, only contact with liver-ECM is not enough to induce complete differentiation.

## 1. Introduction

**Figure 1:**
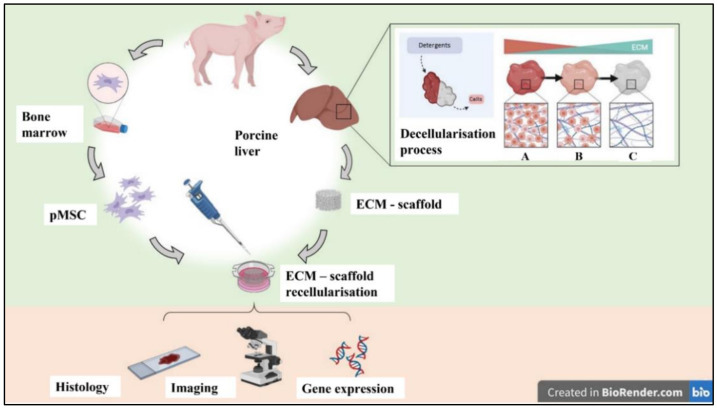
Schematic representation of the experimental design for decellularization/recellularization phases. Bone marrow and porcine liver were collected in sterile conditions under anaesthesia. Liver segments were cut into cubic portions (d = 10 × 10 × 2 mm). The decellularization procedure was performed by 48 h orbicular shacking in sterile 0.15% SDS solution (A = native; B = partially decellularised and C = decellularised). Efficacy evaluation of the decellularisation procedure and ECM integrity were verified by different staining (Haematoxylin/Eosin, Masson’s Trichrome, Picrosirius Red, Alcian Blue and DAPI) and DNA extraction and quantification. Porcine BM samples (20–40 mL) were collected from the posterior iliac crest. Porcine MSC (pMSC) were isolated, expanded and characterized following the minimum criteria of mesenchymal stromal cells. ECM scaffold recellularization with pMSC was performed by static seeding. Cells (1 × 10^6^/50 μL) were seeded drop by drop to cover the liver ECM scaffold. Scaffolds’ repopulation was evaluated by histology, imaging and gene expression.

According to the World Health Organization (WHO) estimates, liver failure-related death have been growing exponentially in recent decades, with an annual increase of 50 million per year [1], and it is regarded as one of the most critical health problems in the world [2,3]. To date, liver transplantation (LT) is widely indicated as the only effective therapy for end-stage liver disease with one-year post-LT survival above 85% [4,5]. However, LT is severely limited by the lack of organ donors. Although much effort has been made to increase the pool of organs to be transplanted, including donation after heart death or pushing the use of marginal livers [6], the need for an LT continues to rise, and the limited liver supply remains the most crucial restrictive driver. In this context, alternative approaches other than LT, as well as “bridge-to-surgery” approaches, are needed. One of the alternative strategies is cellular therapy, such as hepatocyte transplantation (HT). HT has been only used as a bridge-to-surgery solution for more severe patients waiting for an LT, accompanied by relatively modest results and uncertain duration [7,8]. Indeed, HT has rarely generated long-term therapeutic effects since the availability of high-quality hepatocytes is often limited by shortages of donor organs from which cells can be isolated [9,10]. In parallel, mature hepatocytes are difficult to expand in vitro [11]. Therefore, research on alternative options is quickly progressing. Regenerative Medicine (RM) and organ bioengineering (OBE) have been extensively explored to provide transplantable tissues or whole organs with the final goal of creating a three-dimensional (3D) microenvironment mimicking the organ’s native structure [12]. This approach may make the use of autologous cells theoretically possible, eliminating the need for post-transplant immunosuppression. According to the literature, an extracellular matrix (ECM)-based scaffold can be obtained through a decellularisation process [13,14]. The ECM, composed of collagen, fibronectin, laminin and several growth factors, not only represents appropriate mechanical support but also provides a 3D bioactive microenvironment that helps cellular attachment, proliferation and differentiation [15,16,17,18]. Mesenchymal stromal cells (MSCs) are considered a promising cell source in organ bioengineering, able to repopulate ECM-based scaffolds [19,20,21,22]. In fact, the MSCs ability to differentiate into multiple cell types giving rise to cells of all three germinal layers, including hepatocyte-like cells (HLCs), has been reported [10,23,24,25,26]. In the present study, we hypothesise that a decellularised liver scaffold, repopulated with allogenic porcine mesenchymal stromal cells (pMSCs), could activate and promote the differentiation of pMSCs to hepatocyte-like cells (Figure 1).

## 2. Materials and Methods

### 2.1. Animals

Large White six-month-old piglets (*n* = 3; 30 ± 5 Kg BW, mean weight ± SD) from a disease-free barrier breeding facility were housed in fully air-conditioned rooms (24 °C room temperature, 50% relative humidity) for a minimum of seven days and allowed fasted for 12 h before surgery with ad-libitum access to water. The experimental protocol received approval from the National Animal Care and the Institutional Ethics Committee of the University of Pavia. All animal care and procedures were conducted following the guidelines established by Italian and European legislation (Italian Directives 1992/116; European Directives 86/609/EE and 2010-63UE).

### 2.2. Porcine Liver Retrieval

Animals were pre-medicated with an intramuscular injection of tiletamine hydrochloride-zolazepam hydrochloride (10 mg/kg). Subsequently, the marginal vein of the ear was cannulated, and the anaesthesia induction was performed with propofol (0.2 mg/kg EV). Anaesthesia was maintained during surgery with a continuous infusion of propofol (10 mg/kg/h EV). The surgical procedure was carried out in sterile conditions. Through a midline vertical laparotomy, the liver was retracted ventrally and superiorly, and the posterior diaphragmatic attachments of the liver were divided to fully mobilise the organ. The hepatic hilum, the IVC and the aorta were isolated. After an intravenous injection of heparin (100 U/kg), the aorta was clamped just under the diaphragm, and 0.9% NaCl solution was infused in the aortic cannula while the suprahepatic IVC was transected. After 4000 mL perfusion, the liver was removed by sectioning the hilum and divided into lobes. The liver’s segments were placed in sterile organ bags and frozen at −80 °C until further processing. Animals were euthanised with a bolus of pentobarbital 100 mg/Kg IV.

### 2.3. Liver Decellularisation Procedure

Liver segments were thawed at 4 °C for 24 h and then precision-cut into tissue cubic portions sizing 10 × 10 × 2 mm^3^. Samples were transferred into 50 mL tubes, and residual blood was removed by continuous 12 h of orbital shaking (Heidolph, Schwabach, Germany) (120 rpm) in 0.9% NaCl solution added with heparin (5000 UI/mL). Liver specimens decellularisation was then performed by 48 h of orbital shaking in sterile deionised H_2_O (dH_2_O) containing 0.15% Sodium Dodecyl Sulphate (SDS), an anionic detergent (Sigma-Aldrich, Milan, Italy). The solution was changed every 12 h. To remove the detergent, six washes with 0.9% NaCl solution supplemented with 1% amoxicillin/clavulanic acid (Ibigen, Aprilia, Italy) and 1% fluconazole (Bioindustria, Novi Ligure, Italy) were used. Acellular scaffolds were stored in 1% amoxicillin/clavulanic solution at 4 °C. All procedures were performed at room temperature in sterility.

### 2.4. Histological Analysis and DAPI Staining

Efficacy evaluation of the decellularisation procedure as well ECM integrity were verified via Haematoxylin/Eosin (H&E), Alcian Blue, Masson’s Trichrome and Picrosirius Red stainings (all from Bio-Optica, Milan, Italy). Three different native and decellularised fragments from the same liver were fixed in 4% paraformaldehyde (PFA, Sigma-Aldrich) for 24 h at room temperature, rinsed with PBS solution (pH = 7.2), dehydrated with a gradient alcohol series (30%, 50%, 70%, 90% and 100% *v*/*v*), cleared in xylene and horizontally embedded in paraffin. ECM-sections (8 μm) were obtained using a microtome (Leica Biosystems, Nußloch, Germany) and prepared for histological stains. Samples were examined under a light microscope (Axiophot Zeiss, Zukunftspreis, Germany) equipped with a digital camera. To assess the presence of nuclear materials, nuclear-specific 4,6-diamidino-2-phenylindole (DAPI, Sigma-Aldrich) staining was also performed. ECM samples marked with DAPI solution were observed at Confocal Microscope (Leica, Nußloch, Germany).

### 2.5. DNA Residuals Evaluation

Dry decellularised scaffolds (*n* = 5) were weighed and digested using TNE buffer (Tris 10 mM, NaCl 150 mM and EDTA 10 mM) containing Proteinase K (10 mg/mL, Invitrogen, United States) and 20% SDS. After 4 h at 56 °C incubation, TNE-saturated Phenol solution (Sigma-Aldrich) was added to each sample according to the manufacturer’s instructions. Samples were centrifugated, and the aqueous top layer was recovered. An equal amount of phenol-chloroform solution (1:1) was added. After centrifugation, the aqueous top layer was recovered. Phenol-chloroform-isoamyl alcohol (25:24:1; Sigma-Aldrich) was added in equal amounts to each sample and centrifuged at 8000 rpm for 5 min. Ethanol was added, and the solution was kept at 4 °C for 12 h. Samples were centrifugated to remove ethanol. Finally, the extracted DNA was eluted in 50 µL of DNA-free H_2_O and was quantified using spectrophotometric NanoDrop (Thermo Scientific, Milan, Italy). For all the assays, normalisation was performed with respect to native tissue weight.

We applied the same DNA extraction procedure for fresh liver samples (*n* = 5) after dissociation by GentleMACS Dissociator (Miltenyi Biotec, Bologna, Italy) following the manufacturer’s instructions. As a positive control, we considered the DNA obtained by extraction of scaffold added with known DNA concentrations. Positive controls were treated parallel to every dye cycle.

DNA Electrophoresis was used to detect the length of residual DNA fragments present on ECM scaffolds after the decellularisation protocol (*n* = 3). A 2% agarose (Sigma- Aldrich) *w*/*v* gel was prepared, containing TAE buffer (Tris-acetic acid-EDTA, Euroclone, Milan, Italy). DNA samples were mixed with loading Dye (5:1 ratio, Gel Loading Buffer; Sigma Aldrich). Electrophoresis was performed at a voltage gradient of 5 V/cm for 30 min. Fragments were detected by staining the gel with the intercalating dye (SYBR Safe, Invitrogen, Monza, Italy), followed by visualisation/photography under UV light (Syngene Europe, Cambridge, UK). In order to quantify the different DNA fragment base pair (bp) sizes, a DNA ladder was inserted for scaling purposes.

### 2.6. SEM Analysis

ECM scaffolds were washed twice with Sodium Cacodylate Buffer (SCB; 0.1 M, pH 7.4). Scaffolds were fixed with 2% glutaraldehyde (GDA, Sigma-Aldrich) in SCB. After fixation, ECM scaffolds were rinsed with SCB. Critical-point dehydration was carried out using a graded series of ethanol (30%, 50%, 70%, 90% and 100% *v*/*v*). Dehydrated samples were mounted on an aluminium stub and platinum sputtered. They were finally imaged by Field Emission Scanning Electron Microscopes (FE-SEM-Mira3, TESCAN, Roma, Italy) and Scanning Electron Microscopy/Energy Dispersive Spectroscopy SEM/EDS (Zeiss, Zukunftspreis, Germany).

### 2.7. Scaffold Immunogenicity In Vitro Evaluation

The immunogenicity was evaluated after decellularised scaffold lyophilisation by in vitro assay using human peripheral blood mononuclear cells (PBMCs) from one healthy adult subject. Briefly, after ECM enzymatic digestion at 37 °C for 24 h, the solution was lyophilisated (KrosFlo^®^ Research 2i system, Spectrum Laboratories, Milan, Italy) and freeze-dried, obtaining ECM powder that was preserved at −80 °C until use [27]. As a positive control, lyophilised powder from native liver cubic portions was used. 

PBMCs (1 × 10^5^/well) were cultured in triplicate in RPMI (Euroclone) supplemented with 10% FBS (Euroclone) with or without different concentrations (30, 15 and 7.5 mg/mL) of lyophilised ECM and native liver. After five days of incubation, cultures were pulsed with ^3^H-thymidine (37 kBq/well, Perkin Elmer, Waltham, MA, USA) and harvested after 18 h. ^3^H-thymidine incorporation was measured by Topcount (PerkinElmer). Results were expressed as Stimulation Index (SI = counts per minute of PBMC cocultured with lyophilisated powder/counts per minute of PBMC alone). 

### 2.8. Isolation and Expansion of pMSC

Heparinized BM samples (20–40 mL) were collected from the posterior iliac crest using standard BM aspiration kits with a 15-gauge needle (Medax Mod Cage, Mantova, Italy). Porcine mononuclear cells (pMNCs) were isolated and expanded following a previously described protocol [28]. Cultures were propagated in complete medium: D-MEM Low Glucose (Gibco, Milan, Italy) supplemented with 10% Mesencult (Voden, Milan, Italy) until passage 4 (P4).

### 2.9. Characterisation of pMSC

pMSC were characterised by flow cytometry using anti-porcine specific or cross-reactive antibodies as previously described [28]. In particular, anti-CD45 (LifeSpan Biosciences, Seattle, Washington), CD29 (Acris Antibodies, Herford, Germany), anti-CD90, anti-CD11b and anti-CD105 (all from Abcam, Cambridge, UK) were used. Cells were tested for their ability to differentiate into osteoblasts and adipocytes at early passages (P3), as previously described [28]. 

### 2.10. Recellularisation

ECM scaffolds were placed in a 48-well plate and incubated overnight in a complete medium at 37 °C and 5% CO_2_. pMSC (1 × 10^6^/50 μL) were seeded by releasing cells drop by drop on each scaffold and incubated at 37 °C 5% CO_2_ for 30 min to allow cells spread on the samples completely. 1 mL of complete medium was finally added. The medium was changed twice a week. Seeded ECM scaffolds were evaluated by histological staining at different time points (3, 7, 14 and 21 days of culture).

### 2.11. Metabolic Viability Based Assay(MTT)

After recellularisation, cell viability was estimated by 1-(4,5-Dimethylthiazol-2-yl)-3,5-diphenylformazan (MTT) assay (Sigma-Aldrich). After 3, 7, 14 and 21 days, the culture medium was removed, and MTT solution (5 mg/mL in DMEM-Low Glucose) was added to the removed medium and incubated for 4 h at 37 °C 5% CO_2_. Supernatants were removed, and 0.1% HCl in isopropanol was added to dissolve blue formazan crystals. The optical density of the solution was measured at 570 nm by ELISA Microplate Reader (Microplate Reader Model 680, Sunrise, Tecan, Männedorf, Switzerland). The intensity of the blue/purple product is proportional to the number of cells. Four replicates for each time point were performed, and data were expressed as OD mean ± SE. A number of cells (mean ± Standard Error, SE) was extrapolated from a standard curve obtained with 2D culture pMSCs ranging from 1 × 10^6^ to 15 × 10^3^ cells.

### 2.12. Periodic Acid Schiff (PAS) Staining

Repopulated ECM samples were cut in sections (8 µm) for each time-point, fixed in 4% PFA and then stained using Periodic acid Schiff (PAS) staining kit (Bio-Optica) following manufacture instructions. Samples were examined under a light microscope (Axiophot Zeiss, Zukunftspreis, Germany).

### 2.13. RNA Extraction, Retrotrascription and Real-Time PCR 

For each time point, total RNA from seeded scaffolds was extracted using PureZOL (Bio-rad, Hercules, CA, USA). A reverse Transcriptional M-MLV RT kit (Promega, Madison, WI, USA) was applied on 1 μg of RNA to reverse transcribed into complementary DNA (cDNA). cDNA was quantified using NanoDrop (Thermofisher, Milan, Italy). Real-time PCR for albumin (*ALB*), alpha-fetoprotein (*AFP*), cytochrome 450 subfamily 1A1 (*CYP1A1*), 7A1 (*CYP7A1*), 3A29 (*CYP3A29*), CYP3A46 (*CYP3A46*), glucose-6-phosphatase (*G6PC*), hepatocyte nuclear factor 4a (*HNF4α*), multidrug resistance protein 2 (*MRP2*), Serpine 1 (*SERPINA1),* cytokeratin 18 (*Krt18*), desmin (*DES*) and vimentin (*VIM*) was performed (all from Bio-rad). The assay master mix was prepared with iTaq Universal Probes Supermix 2× (Bio-Rad), primer and fluorogenic probes for each gene tested. 100 ng of cDNA per condition was added to each well. Fresh liver samples were used as a positive control.

Real-time PCR was performed on the Real-Time PCR instrument (AB 7500 Standard System). Data analysis was performed by 7500 fast Real-time PCR systems (Applied Biosystems, Waltham, MA, USA). Expression levels for each gene were calculated using the RQ method. The glyceraldehyde 3 phosphate dehydrogenase (*GADPH*) was used as endogenous internal control and hepatocytes calibrator. 

### 2.14. Statistical Analysis

Stata software (Version 16.1, StataCorp, College Station, TX, USA) was used for statistical analysis. Technical replicates were averaged, and all the statistical analyses were performed using independent biological replicates. Quantitative results are reported as means ± standard deviation (SD). Group comparisons were performed using a two-sample Wilcoxon rank-sum (Mann-Whitney) test. Two-tailed *p*-values below 0.05 were considered statistically significant.

## 3. Results

### 3.1. Liver Decellularisation

**Figure 2:**
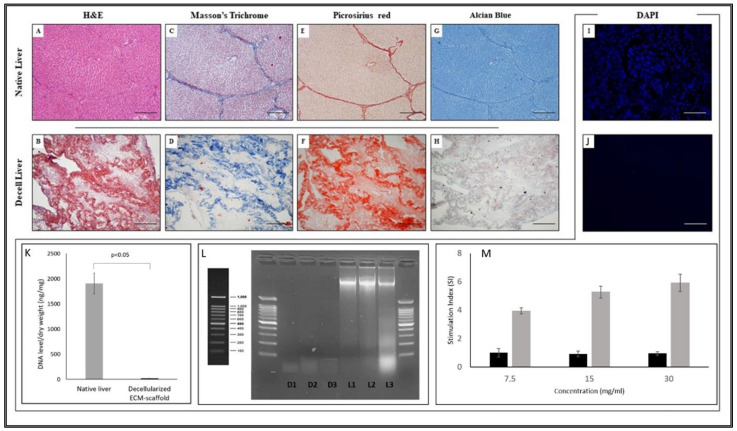
Structural characterisation of porcine liver scaffolds. Panel **A**–**J**. Histological and DAPI staining of native liver and decellularised ECM scaffold. Histological staining revealed the preservation of the ECM structures. In particular, Haematoxylin/Eosin (H&E) (**A**,**B**), Masson’s Trichrome (**C**,**D**) Picrosirius Red (**E**,**F**), and Alcian Blue (**G**,**H**) (magnification 4×). DAPI (**I**,**J**) confirmed the absence of nuclei and residual nuclear material (magnification 10×). Scale bar: 200 µm for each. Panel (**K**). DNA level in native and decellularised scaffolds. Values represent mean ± SE of five samples for each group (*p* < 0.05; *n* = 5). Panel (**L**). DNA Electrophoresis. Fragments were detected by staining the agarose gel with SYBR Green, followed by visualisation/photography under UV light. In order to quantify the different DNA fragment bp sizes, a DNA ladder was inserted for scaling purposes. DNA Electrophoresis on three ECM samples (D1, D2 and D3) showed residual DNA fragments smaller than 100 bp. Panel (**M**). Native liver and decellularised ECM immunogenicity in vitro evaluation. No immunogenic effect was observed at the three different concentrations for decellularised ECM. As a positive control, lyophilised native liver was used at the same concentrations. Results are reported as SI. Values represent the mean ± SD of five replicates. Black bars: lyophilised decellularised ECM; grey bars: lyophilised native liver.

Porcine livers were successfully retrieved using a surgical technique similar to those used for multi-organ explants in a deceased donor. During the decellularisation, the macroscopic appearance of the liver specimens shifted, from dark red to translucent white, confirming the progressive cellular removal. The decellularisation protocol resulted in a complete cell removal, but the preservation of key ECM components was confirmed qualitatively by histological staining. In Figure 2, differences between native and decellularised livers are reported. In particular, standard H&E staining proved the absence of cells and the presence of collagen on liver scaffold-ECM, as well as the preservation of the ECM micro-architecture integrity (Figure 2B vs A). Masson’s Trichrome and PicroSirius red staining revealed the preservation of collagen structures through the portal tracts and vessel walls and the absence of linear collagen fibres in the parenchymal space (Figure 2D and F vs C and E, respectively). By Alcian blue staining, preservation of acid mucins in liver scaffold-ECM was highlighted (Figure 2H vs G). In all samples, it was evident that the structural collapse of the lobular architecture was due to cellular removal. DAPI staining showed the absence of nuclei on the ECM scaffold (Figure 2J vs I). Decellularized scaffold showed a ratio of DNA level/dry weight (mean ± SE) significantly lower than native liver (20.3 ± 1.9 ng/mg and 1907 ± 206.2 ng/mg, respectively; *p* < 0.05) (Figure 2K). Moreover, decellularised samples contained residual DNA fragments smaller than 100 bp (Figure 2L). 

Taken together, and in accordance with criteria defined by Crapo et al. [29], the data confirm that the decellularisation method was able to remove the resident cells whilst overall preserving the biochemical and structural properties of liver ECM. Moreover, to evaluate the immunogenicity of decellularised ECM we set up an in vitro model testing the activation of human PBMCs in the presence of lyophilised ECM or native liver. We could demonstrate that the decellularised ECM produced by our technique did not induce in vitro proliferation of human PBMCs, showing no immunogenic effect (Figure 2M).

### 3.2. 3D Architecture and Ultrastructure

**Figure 3:**
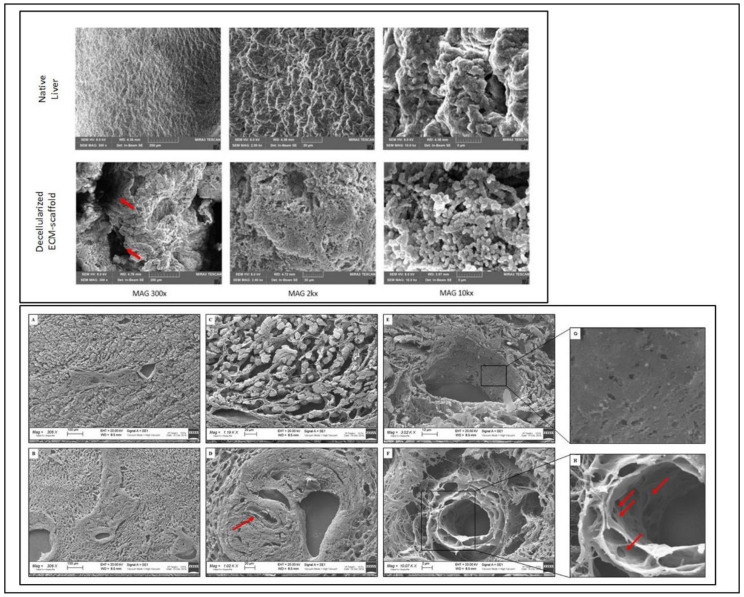
**Upper** panel: Scanning Electron Microscopy (SEM) of the native and decellularised scaffold at different magnifications (300×, 2k× and 10k×). SEM analysis of the acellular ECM showed a rough surface and preserved three-dimensional structures characterised by highly interconnected porosity (arrows = vessel tracts). **Lower** panel (**A**–**F**): SEM images of decellularised ECM-scaffold slides showing the complete cellular removal and the 3D preservation of the ultrastructure after decellularisation (arrows = vessel tracts). Details are highlighted (**G**,**H**).

Scanning electron microscopy of the liver scaffold revealed that our decellularisation protocol preserves hepatic ECM microanatomy and ultrastructure associated with maintaining key hepatic features, including the honeycomb-like arrangement and the presence of an organised network of ECM fibrils of liver lobules (Figure 3). 

### 3.3. Mesenchymal Stromal Cells Isolation and Characterisation 

pMSCs were successfully isolated and expanded in vitro from all porcine BM samples. As already reported [27], cells were plastic adherent and showed the typical spindle-shaped morphology. pMSCs were positive for CD90, CD29, CD105, and negative for CD45 and CD11b and were able to differentiate toward adipogenic and osteogenic lineages. Results are reported as supplementary data in Figure S1.

### 3.4. ECM Scaffold Recellularisation

**Figure 4:**
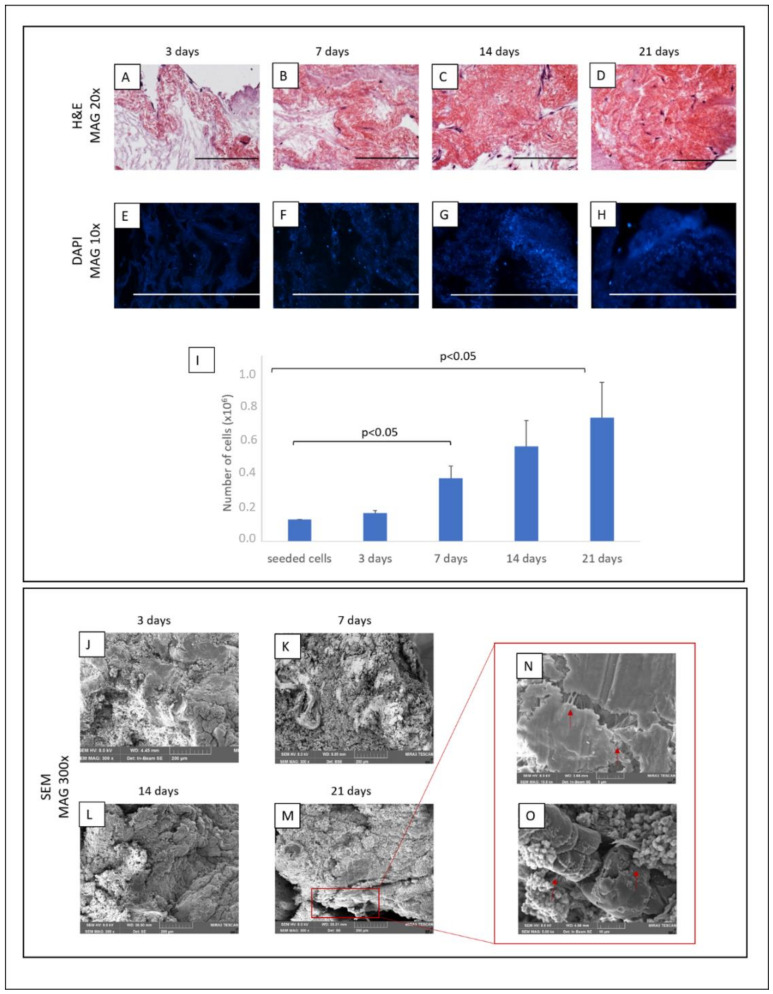
ECM scaffolds repopulated by pMSC. Upper panel: H&E (**A**–**D**) and DAPI (**E**–**H**) staining demonstrated that adherent cells increased progressively, reaching almost a complete scaffold coverage (scale bar: 200 µm for each). The viability of pMSCs seeded on scaffolds was confirmed by MTT assay (**I**). Lower panel (**J**–**O**): The degree of repopulation was also evaluated by SEM analysis. SEM images showed the formation of intracellular junctions and the development of a new extracellular matrix (arrows).

pMSCs were able to adhere, grow and infiltrate the ECM, as demonstrated by H&E and DAPI staining (Figure 4A–H). The MTT assay confirmed that cells on the scaffold increased progressively and remained viable for 21 culture days, reaching an approximate total of 697.000 ± 180.000 cells (mean ± SE), increasing between 5 and6 fold the number of seeded cells (Figure 4I).

We confirmed the presence of pMSCs and their degree of repopulation also by FE-SEM analysis (Figure 4J–M).

Furthermore, no signs of cell suffering as morphological alteration or cell detachment were present after 21 days, suggesting that ECM represents a suitable microenvironment for cell growth and survival. We also could observe the formation of intracellular junctions and the development of a new extracellular matrix, representing a positive step for the recellularisation (Figure 4N,O).

#### Differentiation of pMSCs

**Figure 5:**
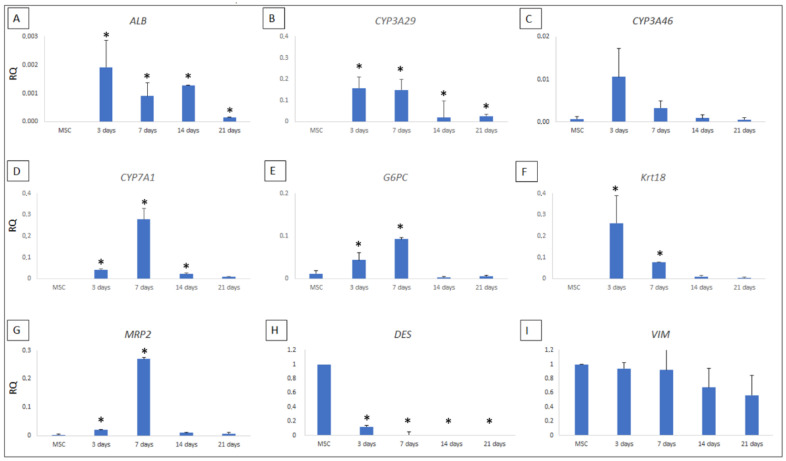
RT-PCR analysis of hepatic genes. The expression levels of the seven hepatic genes showing an increase during the culture on ECM are reported (**A**–**G**). Desmin and Vimentin were used as MSC-specific markers (**H**,**I**). The downregulation of desmin represents the staminality loss. Results were expressed as RQ, normalising the gene expression of interest, with GAPDH as an endogenous internal control. As a calibrator, hepatocytes were used for hepatic-specific genes and pMSC for MSC-specific genes (vimentin and desmin). Results are represented as median ± SE (*n* = 4). * indicate a significant difference 3D vs 2D cultured pMSCs. The exact *p* values are reported in the text.

The expression of 11 genes associated with different phases of hepatic development was compared between 2D and 3D cultured pMSCs. After culture, pMSCs seeded on ECM showed significantly higher levels of *ALB* (*p* < 0.0001 for each timepoint), *CYP3A29* (*p* < 0.0001 for each timepoint), *CYP7A1* (*p* = 0.008, *p* = 0.0002, and *p* = 0.01 at 3, 7 and 14 days, respectively)*, G6PC* (*p* = 0.017and *p* = 0.003, at 3 and 7 days, respectively)*, Krt18* (*p* < 0.005 for 3 and 7 days) *and MRP2* (*p* = 0.01 and *p* = 0.001, at 3 and 7 days, respectively) gene relative expression than in 2D cultured pMSCs evaluated at the same time points (Figure 5A–G). While no significant difference was observed for *CYP3A46*. Additionally, the analysis of MSC-specific markers such as *DES* and *VIM* showed an early and significant decrease of *DES* after three days of culture maintained at each timepoint (*p* < 0.0001 for all). At the same time, a slight reduction was observed for VIM after 21 days of culture (Figure 5H,I).

**Figure 6:**
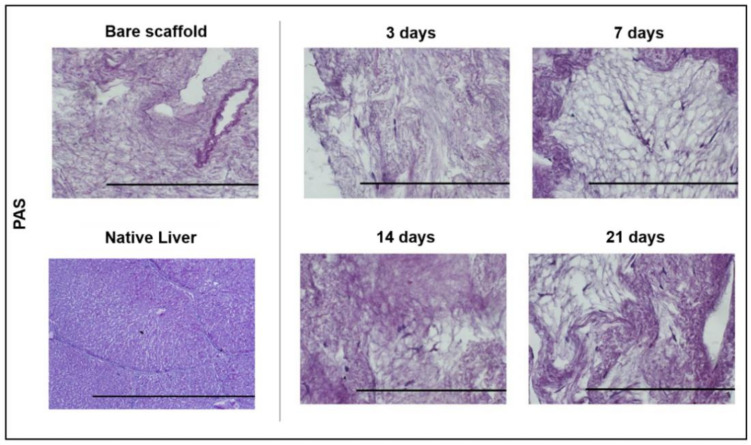
Functional evaluation of pMSC seeded on ECM scaffolds. PAS staining was used to identify glycogen. No deposits of glycogen/polysaccharides that should result in pink/red were observed at the four different time points after PAS staining. In the figure, cells/ECM resulted in dark purple. PAS staining of the native liver is also reported for comparison. (Magnification 40×, scale bar: 200 µm for each).

Moreover, PAS staining for glycogen, as a marker of functional differentiation towards hepatocytes, was performed on the bare and cultured scaffolds at each time point. As shown in Figure 6, no positivity was detected.

## 4. Discussion

Organ transplantation is severely restricted by the lack of donors, the insufficient organ supply the most crucial restrictive driver. OBE has been extensively explored in the last few years to provide transplantable tissues or organs with the final goal of recreating a 3D structure mimicking the native function. In this context, towards the clinical translation, one of the most critical issues to get over is to obtain a relevant-sized hepatic scaffold to repopulate [30]. Different studies show how a 3D-culture condition could improve cellular growth [31,32,33]. To date, the comparison between synthetic or biological scaffolds led controversial results [30]. However, several studies on the extracellular matrix reveal a state of dynamic reciprocity between cells and ECM. It is well reported that the surrounding environment may address cells towards proliferation and differentiation by responding to local signals. 

For ECM-scaffold technology, porcine organs are considered an optimal source. They are very close in size to human counterparts, becoming a good acellular and non-immunogenic 3D surface suitable for repopulating with human cells. In the preclinical setting, three main phases are to be taken into account: the organ decellularisation strategy, the recellularisation process and the interactions between cells and ECM. In this study, we set up a porcine liver decellularisation methodology. Then, we evaluated the feasibility of repopulating ECM with pMSC and the possible influence in promoting the differentiation of pMSC towards hepatocyte-like cells. In literature, specialised decellularisation procedures are developed to remove cellular components by combining physical, chemical and enzymatic methods [34,35,36,37,38]. We optimised a standardised protocol used in our group for kidney decellularisation [39]. A multi-step decellularisation protocol was applied to the organs, including organ freezing/thawing and SDS treatment. It was well demonstrated that a single cycle of freezing/thawing could enhance the detachment of the native cells without damaging the structure of ECM [29]. The second step of decellularisation was based on the use of SDS, a detergent that lyses cell membranes and causes interruption of noncovalent bonds between ECM structural proteins [40]. After this procedure, the evaluation of residual materials within the decellularised scaffolds is mandatory to avoid in vitro cytotoxicity or in vivo adverse host responses during the reintroduction of the organ [12,41,42,43]. In the present study, the presence of nuclear material was qualitatively and quantitatively evaluated after decellularisation, resulting in compliance with the reported criteria [29]. By histological and DAPI staining, no evidence of cellular nuclei was found, and an intact cellular matrix was observed. The decellularised scaffold displayed very small quantities of DNA fragments, compatible with an optimal decellularisation process, with only 3.5% of the total DNA extracted from the native liver still present. Moreover, by SEM, the decellularised scaffold appeared as a rough surface, with a preserved three-dimensional network of the vascular structures of the native organ. In some points, it was also possible to observe some gaps compatible with a single hepatocyte dimension. Thus, the preserved continuity of the ECM and the absence of alterations suggested that the ECM architecture was maintained, giving a 3D structure adequate for the recellularisation phase.

In literature, different stem cells have been proposed as an ideal cell source for scaffold repopulation [19,20]. Among these, MSCs have been taken into account thanks to their ability to differentiate in different lineages, even into hepatocyte-like cells [23,24,25,44,45,46]. In our study, we proceeded to recellularisation using MSCs isolated from porcine BM. The ability of pMSCs to grow on ECM scaffolds was evaluated qualitatively and quantitatively at different time points, concluding that pMSCs, and in general MSC, may represent a good cell population for the recellularisation of ECM scaffolds.

In liver-specific regenerative medicine, the maintenance and gain of HLCs functions are a significant challenge. Recent studies in organ bioengineering have shown that the preservation of ECM induces stem cells to differentiate into tissue-specific cells. Several approaches have been investigated in the literature, such as different cell sources, types of scaffolds and induction protocols [47,48,49,50]. In this regard, although the role of ECM in the hepatic maturation of induced pluripotent stem cells (iPSCs) was not fully understood, Park et al. [51] have shown that liver ECM, in addition to specific growth factors, may enhance liver cells development and maturation. Li et al. [31] demonstrated that the presence of rat ECM increased hepatic gene expression in HLCs derived from human MSC. Kim et al. [50] developed a liver-specific gene expression panel algorithm that defines the differentiation status or similarity degree between liver and differentiated cultured cells. 

In order to assess the differentiation potential due to only ECM, we evaluated the pMSC gene expression of different liver-specific markers without adding exogenous stimuli. We selected 13 genes based on data reported in the literature evaluating the decellularised ECM induction of MSCs towards hepatocyte-like cells [26,31,51,52,53]. 

We observed that only contact with liver-specific ECM was insufficient to induce a complete differentiation of pMSCs in HLCs. However, we observed after culture a transient upregulation of hepatic markers, validated by the finding of downregulation of *DES* expression, considered an MSC staminality marker. We believe that only liver ECM gives an “input” toward hepatocyte differentiation; however, it is not enough to obtain a complete HLC maturation. We know that the expression levels of liver-specific lineage markers cannot provide complete information regarding cell differentiation status. We could consider a limit of the present study, the lack of protein quantification and/or immunohistochemical staining that could provide more reliable results. Since we did not reach a complete differentiation of pMSCs toward HLCs, due to the only ECM scaffold, we cannot postulate an action mechanism. However, as reported in the literature, we believe that the structure and composition of the ECM favour the differentiation of cells through direct contact with organ specific-matrix by an acellular network of macromolecules influencing cellular activities, tissue properties and functions. Our results, pointing out the fundamental role of ECM, support the need for specific stimuli in addition to ECM-seeded cells in order to lead to complete differentiation. 

## 5. Conclusions

The standardisation of scaffold production and ECM repopulation represent crucial steps in the progression of OBE. The present study, investigating if acellular liver ECM alone could influence/promote differentiation of pMSCs toward hepatocyte-like cells, supports the role of ECM in organ reconstitution. We believe that our work presents some novelties. We described a “simple and reproducible” approach to produce an acellular scaffold that effectively decellularised and preserved ECM micro-architecture and bioactive components. Since MSCs are well-known for their proliferation and differentiation abilities in vitro and can promote liver regeneration, we have focused on this type of cells. Access to an abundant, high-quality supply of hepatocytes with therapeutic potential for cell transplantation and extracorporeal support for patients with liver failure is an important issue. We can postulate that our results may provide new insights toward a better understanding of cell differentiation using ECM scaffolds, paving the way towards clinical translation.

## Data Availability

Data used for this manuscript are available on request.

## Supplementary Materials

The following supporting information can be downloaded at: https://www.mdpi.com/article/10.3390/biomedicines10112817/s1, Figure S1: Characterisation of ex-vivo expanded pMSCs.

## References

[1] Murray C.J.L., Lopez A.D. (2013). Measuring the global burden of disease. N. Engl. J. Med..

[2] Sarin S.K., Choudhury A. (2016). Acute-on-chronic liver failure: Terminology, mechanisms and management. Nat. Rev. Gastro. Hepat..

[3] Bernal W., Auzinger G., Dhawan A., Wendon J. (2010). Seminar Acute liver failure. Lancet.

[4] Kwong A., Kim W.R., Lake J.R., Smith J.M., Schladt D.P., Skeans M.A., Noreen S.M., Foutz J., Miller E., Snyder J.J. (2020). OPTN/SRTR 2018 Annual Data Report: Liver. Am. J. Transplant..

[5] U.S. Department of Health and Human Services (2014). OPTN/SRTR 2012 Annual Data Report—United States Organ Transplantation. Am. J. Transplant..

[6] Merion R.M., Pelletier S.J., Goodrich N., Englesbe M.J., Delmonico F.L. (2006). Donation after cardiac death as a strategy to increase deceased donor liver availability. Ann. Surg..

[7] Bilir B., Guinette D., Karrer F., Kumpe D.A., Krysl J., Stephens J., McGavran L., Ostrowska A., Durham J. (2000). Hepatocyte transplantation in acute liver failure. Liver Transplant..

[8] Nussler A., Konig S., Ott M., Sokal E., Christ B., Thasler W., Brulport M., Gabelein G., Schormann W., Schulze M. (2006). Present status and perspectives of cell-based therapies for liver diseases. J. Hepatol..

[9] O’Donoghue K., Fisk N.M. (2004). Fetal stem cells. Best Pract. Res. Clin. Obstet. Gynaecol..

[10] Nicolas C., Wang Y., Luebke-Wheeler J., Nyberg S.L. (2016). Stem cell therapies for treatment of liver disease. Biomedicines.

[11] Ibars E.P., Cortes M., Tolosa L., Gómez-Lechón M.J., López S., Castell J.V., Mir J. (2016). Hepatocyte transplantation program: Lessons learned and future strategies. World J. Gastroenterol..

[12] Badylak S.F., Taylor D., Uygun K. (2010). Whole Organ Tissue Engineering: Decellularization and Recellularization of Three-Dimensional Matrix Scaffolds. Annu. Rev. Biomed. Eng..

[13] Baptista P.M., Orlando G., Mirmalek-Sani S.H., Siddiqui M., Atala A., Soker S. (2009). Whole organ decellularization - A tool for bioscaffold fabrication and organ bioengineering. Annu. Int. Conf. IEEE Eng. Med. Biol. Soc..

[14] Uygun B.E., Soto-Gutierrez A., Yagi H., Izamis M.L., Guzzardi M.A., Shulman C., Milwid J., Kobayashi N., Tilles A., Berthiaume F. (2010). Organ reengineering through development of a transplantable recellularized liver graft using decellularized liver matrix. Nat. Med..

[15] Abazari M.F., Soleimanifar F., Enderami S.E., Nasiri N., Nejati F., Mousavi S.A., Soleimani M., Kiani J., Ghoraeian P., Kehtari M. (2020). Decellularized amniotic membrane Scaffolds improve differentiation of iPSCs to functional hepatocyte-like cells. J. Cell Biochem..

[16] Meyer S.R., Nagendran J., Desai L.S., Rayat G.R., Churchill T.A., Anderson C.C., Rajotte R.V., Lakey J.R., Ross D.B. (2005). Decellularization reduces the immune response to aortic valve allografts in the rat. J. Thorac. Cardiovasc. Surg..

[17] Sánchez-Romero N., Sainz-Arnal P., Pla-Palacín I., Dachary P.R., Almeida H., Pastor C., Soto D.R., Rodriguez M.C., Arbizu E.O., Martinez L.B. (2019). The role of extracellular matrix on liver stem cell fate: A dynamic relationship in health and disease. Differentiation.

[18] Fitzpatrick E., Mitry R.R., Dhawan A. (2009). Human hepatocyte transplantation: State of the art. J. Int. Med..

[19] Lorvellec M., Scottoni F., Crowley C., Fiadeiro R., Maghsoudlou P., Pellegata A.F., Mazzacuva F., Gjinovci A., Lyne A.M., Zulini J. (2017). Mouse decellularised liver scaffold improves human embryonic and induced pluripotent stem cells differentiation into hepatocyte-like cells. PLoS ONE.

[20] Wang B., Jakus A.E., Baptista P.M., Soker S., Soto-Gutierrez A., Abecassis M.M., Shah R.N., Wertheim J.A. (2016). Functional Maturation of Induced Pluripotent Stem Cell Hepatocytes in Extracellular Matrix-A Comparative Analysis of Bioartificial Liver Microenvironments. Stem Cells Transl. Med..

[21] Banas A., Yamamoto Y., Teratani T., Ochiya T. (2007). Stem cell plasticity: Learning from hepatogenic differentiation strategies. Dev. Dynam..

[22] Panta W., Imsoonthornruksa S., Yoisungnern T., Suksaweang S., Ketudat-Cairns M., Parnpai R. (2019). Enhanced hepatogenic differentiation of human wharton’s jelly-derived mesenchymal stem cells by using three-step protocol. Int. J. Mol. Sci..

[23] Al Ghrbawy N.M., Afify R.A.A.M., Dyaa N., El Sayed A.A. (2016). Differentiation of Bone Marrow: Derived Mesenchymal Stem Cells into Hepatocyte-like Cells. Indian J. Hematol. Blood Transfus..

[24] Xie P.Y., Hu X.J., Guo R.M., Meng X.C., Pang P.F., Zhou Z.Y., Li D., Shan H. (2019). Generation of functional hepatocyte-like cells from human bone marrow mesenchymal stem cells by overexpression of transcription factor HNF4α and FOXA2. Hepatob. Pancreat. Dis. Int..

[25] Zhou X., Cui L., Zhou X., Yang Q., Wang L., Guo G., Hou Y., Cai W., Han Z., Shi Y. (2017). Induction of hepatocyte-like cells from human umbilical cord-derived mesenchymal stem cells by defined microRNAs. J. Cell Mol. Med..

[26] Najimi M., Khuu D.N., Lysy P.A., Jazouli N., Abarca J., Sempoux C., Sokal E.M. (2007). Adult-derived human liver mesenchymal-like cells as a potential progenitor reservoir of hepatocytes?. Cell Transplant..

[27] Bari E., Di Silvestre D., Mastracci L., Grillo F., Grisoli P., Marrubini G., Nardini M., Mastrogiacomo M., Sorlini M., Rossi R. (2020). GMP-compliant sponge-like dressing containing MSC lyo-secretome: Proteomic network of healing in a murine wound model. Eur. J. Pharm. Biopharm..

[28] Comite P., Cobianchi L., Avanzini M.A., Zonta S., Mantelli M., Achille V., De Martino M., Cansolino L., Ferrari C., Alessiani M. (2010). Isolation and Ex Vivo Expansion of Bone Marrow-Derived Porcine Mesenchymal Stromal Cells: Potential for Application in an Experimental Model of Solid Organ Transplantation in Large Animals. Transplant. Proc..

[29] Crapo P.M., Gilbert T.W., Badylak S.F. (2011). An overview of tissue and whole organ decellularization processes. Biomaterials.

[30] Croce S., Peloso A., Zoro T., Avanzini M.A., Cobianchi L. (2019). A Hepatic Scaffold from Decellularized Liver Tissue: Food for Thought. Biomolecules.

[31] Li Y., Wu Q., Wang Y., Li L., Chen F., Shi Y., Bao J., Bu H. (2017). Construction of bioengineered hepatic tissue derived from human umbilical cord mesenchymal stem cells via aggregation culture in porcine decellularized liver scaffolds. Xenotransplantation.

[32] Luo Y., Lou C., Zhang S., Zhu Z., Xing Q., Wang P., Liu T., Liu H., Li C., Shi W. (2018). Three-dimensional hydrogel culture conditions promote the differentiation of human induced pluripotent stem cells into hepatocytes. Cytotherapy.

[33] Rajendran D., Hussain A., Yip D., Parekh A., Shrirao A., Cho C.H. (2017). Long-term liver-specific functions of hepatocytes in electrospun chitosan nanofiber scaffolds coated with fibronectin. J. Biomed. Mater. Res..

[34] Mazza G., Rombouts K., Rennie Hall A., Urbani L., Vinh Luong T., Al-Akkad W., Longato L., Brown D., Maghsoudlou P., Dhillon A.P. (2015). Decellularized human liver as a natural 3D-scaffold for liver bioengineering and transplantation. Sci. Rep..

[35] Verstegen M.M.A., Willemse J., van den Hoek S., Kremers G.J., Luider T.M., van Huizen N.A., Willemssen F.E.J.A., Metselaar H.J., IJzermans J.N.M., van der Laan L.J.W. (2017). Decellularization of Whole Human Liver Grafts Using Controlled Perfusion for Transplantable Organ Bioscaffolds. Stem Cells Dev..

[36] Yagi H., Fukumitsu K., Fukuda K., Kitago M., Shinoda M., Obara H., Itano O., Kawachi S., Tanabe M., Coudriet G.M. (2013). Human-Scale Whole-Organ Bioengineering for Liver Transplantation: A Regenerative Medicine Approach. Cell Transplant..

[37] Moulisová V., Jiřík M., Schindler C., Červenková L., Pálek R., Rosendorf J., Arlt J., Bolek L., Šůsová S., Nietzsche S. (2020). Novel morphological multi-scale evaluation system for quality assessment of decellularized liver scaffolds. J. Tissue Eng..

[38] Naeem E.M., Sajad D., Talaei-Khozani T., Khajeh S., Azarpira N., Alaei S., Tanideh N., Reza T.M., Razban V. (2019). Decellularized liver transplant could be recellularized in rat partial hepatectomy model. J. Biomed. Mater. Res. A..

[39] Peloso A., Ferrario J., Maiga B., Benzoni I., Bianco C., Citro A., Currao M., Malara A., Gaspari A., Balduini A. (2015). Creation and implantation of acellular rat renal ECM-based scaffolds. Organogenesis.

[40] Liu Z.Z., Wong M.L., Griffiths L.G. (2016). Effect of bovine pericardial extracellular matrix scaffold niche on seeded human mesenchymal stem cell function. Sci. Rep..

[41] Zhang J., Zhao X., Liang L., Li J., Demirci U., Wang S.Q. (2018). A decade of progress in liver regenerative medicine. Biomaterials.

[42] Keane T.J., Londono R., Turner N.J., Badylak S.F. (2012). Consequences of ineffective decellularization of biologic scaffolds on the host response. Biomaterials.

[43] Alaby Pinheiro Faccioli L., Suhett Dias G., Hoff V., Lemos Dias M., Ferreira Pimentel C., Hochman-Mendez C., Braz Parente D., Labrunie E., Souza Mourão P., Rogério de Oliveira Salvalaggio P. (2020). Optimizing the Decellularized Porcine Liver Scaffold Protocol. Cells Tissues Organs.

[44] Brückner S., Tautenhahn H.M., Winkler S., Stock P., Dollinger M., Christ B. (2014). A fat option for the pig: Hepatocytic differentiated mesenchymal stem cells for translational research. Exp. Cell Res..

[45] Ghaedi M., Soleimani M., Shabani I., Duan Y., Lotfi A.S. (2012). Hepatic differentiation from human mesenchymal stem cells on a novel nanofiber scaffold. Cell Mol. Biol. Lett..

[46] Soto-Gutierrez A., Zhang L., Medberry C., Fukumitsu K., Faulk D., Jiang H., Reing J., Gramignoli R., Komori J., Ross M. (2011). A Whole-Organ Regenerative Medicine Approach for Liver Replacement. Tissue Eng. Part C Methods.

[47] Bao J., Wu Q., Wang Y., Li Y., Li L., Chen F., Wu X., Xie M., Bu H. (2016). Enhanced hepatic differentiation of rat bone marrow-derived mesenchymal stem cells in spheroidal aggregate culture on a decellularized liver scaffold. Int. J. Mol. Med..

[48] Coronado R.E., Somaraki-Cormier M., Natesan S., Christy R.J., Ong J.L., Halff G.A. (2017). Decellularization and Solubilization of Porcine Liver for Use as a Substrate for Porcine Hepatocyte Culture: Method Optimization and Comparison. Cell Transplant..

[49] Jaramillo M., Yeh H., Yarmush M.L., Uygun B.E. (2018). Decellularized human liver extracellular matrix (hDLM)-mediated hepatic differentiation of human induced pluripotent stem cells (hIPSCs). J. Tissue Eng. Regen. Med..

[50] Kim D.S., Ryu J.W., Son M.Y., Oh J.H., Chung K.S., Lee S., Lee J.J., Ahn J.H., Min J.S., Ahn J. (2017). A liver-specific gene expression panel predicts the differentiation status of in vitro hepatocyte models. Hepatology.

[51] Park K.M., Hussein K.H., Hong S.H., Ahn C., Yang S.R., Park S.M., Kweon O.K., Kim B.M., Woo H.M. (2016). Decellularized Liver Extracellular Matrix as Promising Tools for Transplantable Bioengineered Liver Promotes Hepatic Lineage Commitments of Induced Pluripotent Stem Cells. Tissue Eng..

[52] Ji R., Zhang N., You N., Li Q., Liu W., Jiang N., Liu J., Zhang H., Wang D., Tao K. (2012). The differentiation of MSCs into functional hepatocyte-like cells in a liver biomatrix scaffold and their transplantation into liver-fibrotic mice. Biomaterials.

[53] Fráguas-Eggenschwiler M., Eggenschwiler R., Söllner J.H., Cortnumme L., Vondran F.W.R., Cantz T., Ott M., Niemann H. (2021). Direct conversion of porcine primary fibroblasts into hepatocyte-like cells. Sci. Rep..

