# Supplementary material for: Mesenchymal Stromal Cell on Liver Decellularised Extracellular Matrix for Tissue Engineering
Source: Biomedicines. 2022 Nov 4;10(11):2817. doi: 10.3390/biomedicines10112817 (PMC9687774; doi:10.3390/biomedicines10112817)
Supplement: Supplementary file 1 [file biomedicines-10-02817-s001.zip › biomedicines-1953678-supplementary.pdf]

Supplementary material

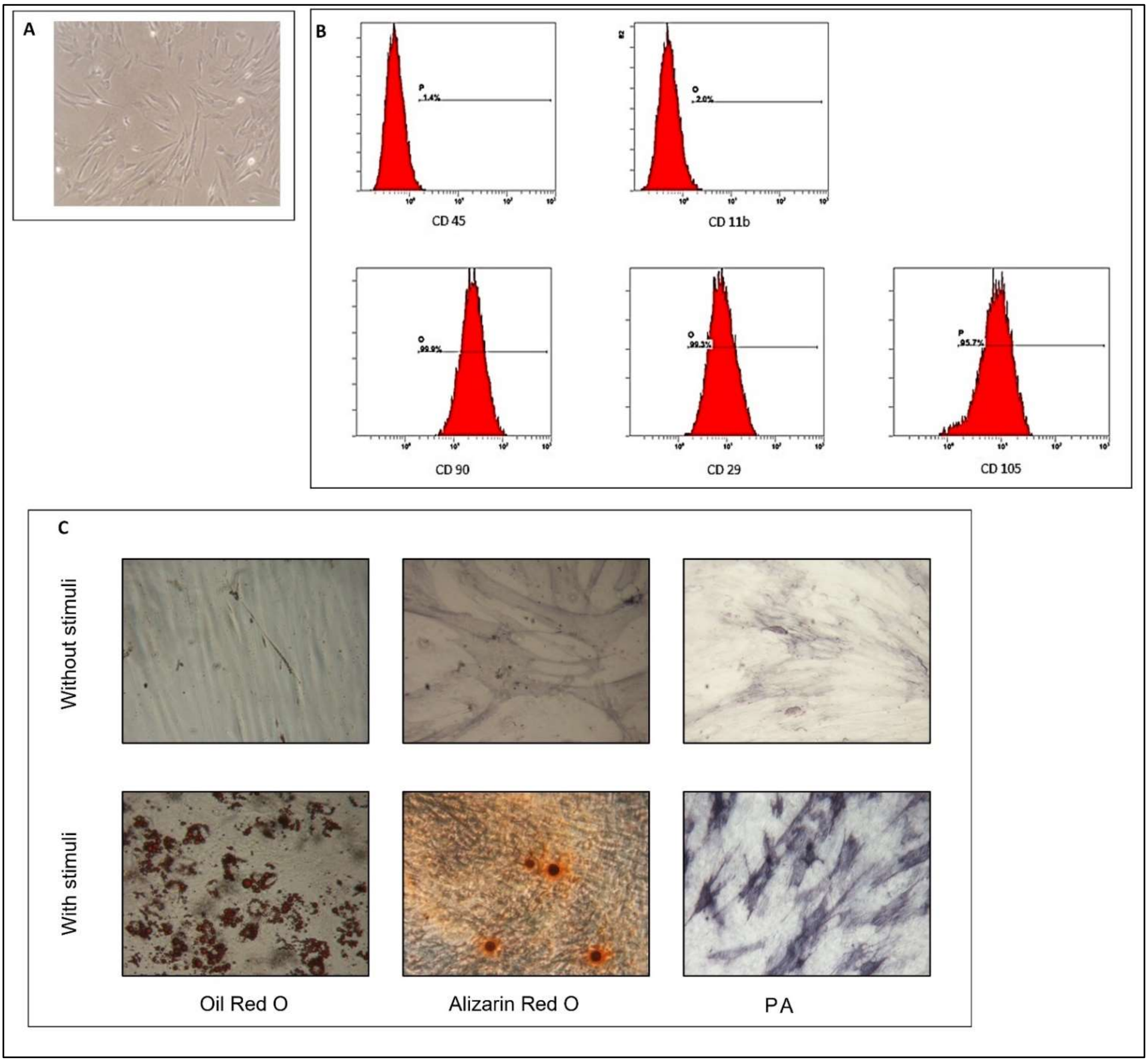

Figure S1. Characterisation of ex-vivo expanded pMSCs. Panel A： typical "spindle-shape" morphology (magnification 10×). Panel B： Immunophenotypical expression of pMSCs. Expression of the typical surface markers CD90, CD29 and CD105 and absence of CD45 and CD11b. Panel C： Adipogenic and osteogenic in vitro differentiation capacity: the differentiation into adipocytes is revealed by the formation of lipid droplets stained with Oil red O and the differentiation into osteoblasts is demonstrated by the presence of Alkaline phosphatase (PA) activity and by the histological detection of calcium depositions positive for Alizarin Red S (magnification 20×).
